# Three-dimensional assessment of mandibular asymmetry in patients with unilateral cleft lip and palate at different growth stages: a cross-sectional study

**DOI:** 10.1590/2177-6709.29.6.e242496.oar

**Published:** 2024-12-16

**Authors:** Thaís de Lima AZEREDO, Laíze Rosa Pires FREITAS, Rodrigo Villamarim SOARES, Dauro Douglas OLIVEIRA, Maria Augusta VISCONTI, Eduardo Murad VILLORIA

**Affiliations:** 1 Universidade Federal do Rio de Janeiro, Faculdade de Odontologia, Departamento de Patologia e Diagnóstico Oral (Rio de Janeiro, RJ, Brazil).; 2 Private practice (Belo Horizonte/MG, Brazil).; 3 Potifícia Universidade Católica de Minas Gerais, Departamento de Odontologia (Belo Horizonte, MG, Brazil).; 4 Potifícia Universidade Católica de Minas Gerais, Departamento de Ortodontia (Belo Horizonte, MG, Brazil).

**Keywords:** Cleft lip and palate, Mandible, Facial asymmetry, Maxillofacial development, Cone-beam computed tomography, Fissura labiopalatina, Mandíbula, Assimetria facial, Desenvolvimento maxilofacial, Tomografia computadorizada de feixe cônico

## Abstract

**Introduction:**

The early diagnosis of mandibular asymmetry (MA) in patients with unilateral cleft lip and palate (UCLP) can contribute to its treatment.

**Objective:**

The aim of this study was to evaluate the occurrence and the extent of MA in UCLP patients at different growth stages.

**Methods:**

Cone-beam computed tomography (CBCT) of 47 UCLP patients were included, and divided into two groups (prepubertal stage and pubertal stage). The mandibular ramus height (Co-Go), mandibular body length (Go-Me), total mandibular length (Co-Me), gonial angle (CoGoMe), the volume of the mandibular condyles, and lateral chin deviation were evaluated. The cleft side (CS) and noncleft side (NCS) were compared using the paired *t*-test for dependent samples. Chin deviation and its possible association with specific sides were evaluated using the Mann-Whitney and Fisher’s Exact tests, respectively. The significance level was set at 5%.

**Results:**

Comparison of CS and NCS revealed that in prepubertal stage, Co-Me was shorter (105.5 ± 5.7 mm; *p* = 0.036) in the CS, and that in pubertal stage, Co-Go was lower (46 ± 6 mm; *p*= 0.004) and Go-Me was greater (78.5 ± 5.8 mm; *p* = 0.026) in the CS. In both growth stages, a significant association (*p*< 0.05) was observed between the side to which the chin has deviated and the CS.

**Conclusions:**

Although patients with UCLP in the prepubertal and pubertal stages presented significant measurements revealing MA, only Co-Go in the pubertal stage showed a clinically relevant difference. This specific result indicates that MA must be monitored during patients’ growth.

## RESUMO

**Introdução:**

O diagnóstico precoce da assimetria mandibular (AM) em pacientes com fissura labiopalatina unilateral (FLPU) pode contribuir para seu tratamento.

**Objetivo:**

O objetivo deste estudo foi avaliar a ocorrência e a extensão da AM em pacientes com FLPU em diferentes estágios do crescimento.

**Métodos:**

Tomografias computadorizadas de feixe cônico de 47 pacientes com FLPU foram incluídas e divididas em dois grupos (estágio pré-puberal e estágio puberal). A altura do ramo mandibular (Co-Go), comprimento do corpo mandibular (Go-Me), comprimento mandibular total (Co-Me), ângulo goníaco (CoGoMe), volume dos côndilos mandibulares e desvio lateral do mento foram avaliados. Os lados com fissura (LCF) e sem fissura (LSF) foram comparados por meio do teste *t* pareado para amostras dependentes. O desvio do mento e sua possível associação com os lados foram avaliados por meio dos testes Mann-Whitney e Exato de Fisher. O nível de significância foi estabelecido em 5%.

**Resultados:**

No estágio pré-puberal, o Co-Me foi menor (105,5 ± 5,7 mm; *p* = 0,036) no LCF, e no estágio puberal, o Co-Go foi menor (46 ± 6 mm; *p* = 0,004) e o Go-Me foi maior (78,5 ± 5,8 mm; *p* = 0,026) no LCF. Em ambos os estágios do crescimento, foi observada uma associação significativa (*p*< 0,05) entre o lado para o qual o mento estava desviado e o LCF.

**Conclusões:**

Embora pacientes com FLPU nos estágios pré-puberal e puberal tenham apresentado medidas significativas revelando AM, apenas Co-Go no estágio puberal demonstrou uma diferença clinicamente relevante entre os lados. Esse resultado indica que a AM deve ser monitorada durante o crescimento dos pacientes com FLPU.

## INTRODUCTION

There is currently a high global prevalence of patients with cleft lip and palate, and health system policymakers need to take precautionary measures to reduce its incidence, as well as diagnostic and therapeutic measures to reduce the effects of this malformation in children.^1^ Patients affected by cleft lip and palate have significant limitations that interfere with the maxillofacial growth and development.^2^

In patients with unilateral cleft lip and palate (UCLP), mandibular asymmetry, anterior and posterior crossbite, midface deficiency, and Angle Class III malocclusion are usually present.^3^ Mandibular asymmetry (MA) may be associated with UCLP, maxillary atresia, and temporomandibular disorders.^4^ ^-^ ^6^ MA has been described as any dimensional alteration in size, shape, and volume between mandibular hemiarchs,^7^ and is more prevalent in patients with UCLP, when compared with patients with bilateral CLP or without craniofacial malformation.^8^

The skeletal alterations are identified by extra and intraoral evaluations, and confirmed with radiographic or tomographic exams. Different measurement methods have been used to evaluate MA in UCLP patients.^5^ ^,^ ^8^ ^-^ ^11^ In this regard, cone-beam computed tomography (CBCT) is considered the gold standard method for diagnosis and surgical treatment planning of CLP patients,^12^ due to its high resolution and images without overlapping nor distortion. Furthermore, its use has previously been described in different studies,^5^ ^,^ ^8^ ^-^ ^11^ and the development of post-processing software has allowed advances in three-dimensional evaluations of the maxillomandibular morphology of patients with craniofacial deformities, and assessment of their facial growth.^12^ ^-^ ^14^

In addition to the evaluation of chronological age, radiographic and tomographic analysis can be used to assess skeletal maturation, thereby contributing to more precise orthodontic treatment planing.^15^ ^,^ ^16^ However, considering that the growth spurt is influenced by age, gender, ethnicity, genetic factors, and socioeconomic issues,^16^ ^-^ ^18^ bone maturation must be analyzed in imaging exams to define the growth stages (prepubertal, pubertal, and post-pubertal). The cervical vertebrae maturation stage (CVMS) can be evaluated in diagnostic exams by facial imaging, such as lateral cephalometry or CBCT that have previously been performed in patients undergoing orthodontic treatment.^19^ Therefore, additional radiographic exams, such as carpal radiography, are unnecessary, according to the principles of ALARA (as low as reasonably achievable) and ALADAIP (as low as diagnostically acceptable, indication-oriented, and patient-specific).^20^

To date, no previous studies comparing prepubertal and pubertal stages to evaluate MA in patients with UCLP have been observed in the literature. Therefore, considering the importance of CBCT for the three-dimensional skeletal evaluation and identification of the individual’s bone age, and that the early diagnosis of MA in patients with UCLP could, in principle, contribute to their treatment and rehabilitation,^8^ the aim of this study was to evaluate the MA in patients with UCLP in different growth stages.

## MATERIAL AND METHODS

This cross-sectional study was previously approved by the Institutional Review Board of *Universidade Federal do Rio de Janeiro* (protocol #5,377,757) and *Pontifícia Universidade Católica de Minas Gerais* (protocol #5,898,761), and was performed according to the STROBE guidelines for observational studies.^21^ CBCT scans of 52 patients with UCLP treated in the Graduate Program in Orthodontics between 2010 and 2018 were selected for the present study. All CBCT scans were performed before the orthodontic treatment using an i-CAT Classic device (Imaging Sciences International, Hatfield, PA, USA) with the following acquisition parameters: 36 mAs, 120 kVp, a field of view (FOV) of 21 cm x 17 cm, isotropic voxel of 0.3 mm and exposure time of 40 seconds.

The inclusion criteria were: (1) CBCT of patients with complete UCLP; (2) absence of previous orthodontic treatment; (3) history of primary surgical intervention, but no secondary procedure, such as bone grafting; (4) CVMS between 1 and 4. The exclusion criteria were: (1) CBCT of patients with UCLP other than the transforaminal type (complete); (2) patients with bilateral cleft lip and palate or any other craniofacial disorder; (3) CBCT scans with signs of alveolar bone loss and/or intraosseous pathologies with cortical expansion or disruption; (4) CVMS above 4.

Two previously trained and calibrated examiners performed all analyses individually. After 15 days, 20% of the sample was reassessed.^22^ To determine the CVMS and organize the groups into prepubertal (CVMS1-CVMS2) and pubertal stages (CVMS3-CVMS4), the CBCT scans were imported into the Dolphin software (Dolphin Imaging & Management Solutions, Chatsworth, USA) and the images were analyzed in a sagittal view.^23^

The MA analysis was performed using the free and open-source ITK-SNAP software (version 3.8.0, www.itksnap.org), and the 3D Slicer software (version 4.8.1, www.Slicer.org). Initially, the DICOM files were imported into ITK-SNAP for contrast and density adjustment, and conversion to gipl.gz. The CBCT scans in gipl.gz were imported into the 3D Slicer for automatic segmentation of facial bones, construction of 3D virtual models, and head orientation in the Cartesian plane, as described in previous studies.^24^

**Figure 1:**
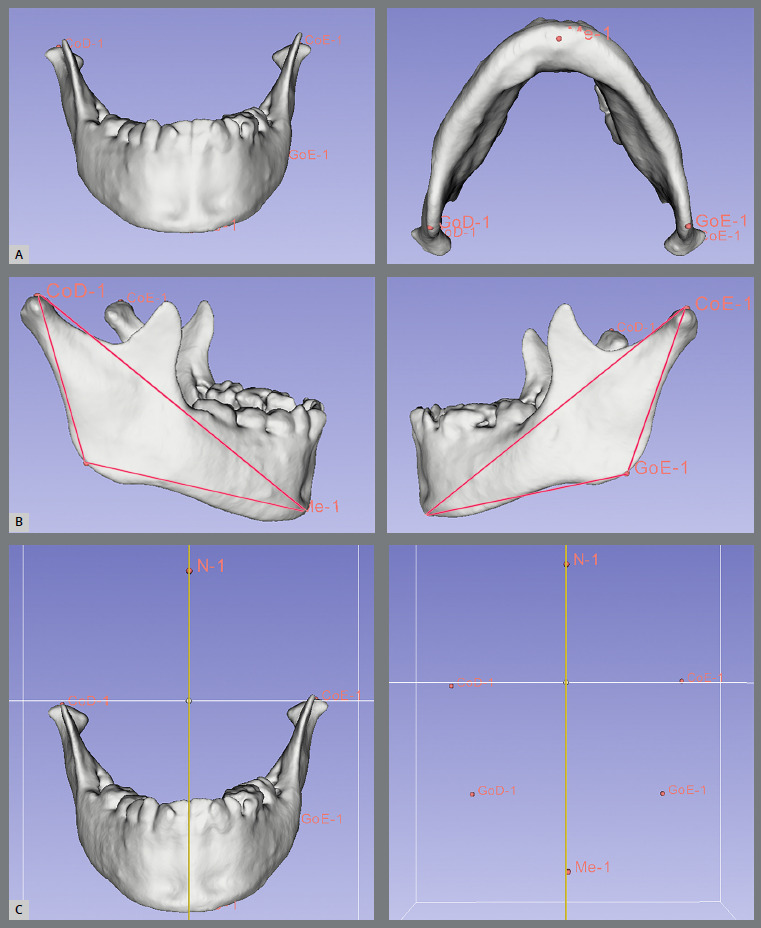
Virtual models in 3D Slicer software. A) Fiducial points applied on the landmarks, B) 3D linear distances and gonial angle, C) evaluation of lateral chin deviation using Me and N as a reference.

The oriented CBCT scan file was imported into the ITK-SNAP for marking the following landmarks: condyle (Co), gonion (Go), menton (Me), and nasion (N). The landmarks were imported into the 3D Slicer, and the fiducial points were applied to measure 3D Euclidean distances (mm), angular measurements, and assessment of lateral chin deviation. The ramus height (Co-Go), the body length (Go-Me), the total mandibular length (Co-Me), and the gonial angle (CoGoMe) were measured bilaterally. The chin deviation was evaluated based on the lateral distance (right-left) between Me and N, which coincides with the median sagittal plane (Fig 1).

**Figure 2:**
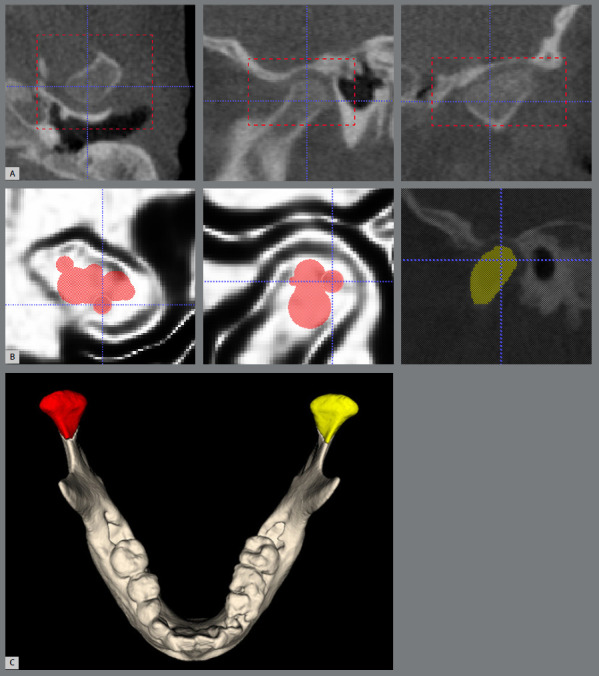
CBCT scans imported in ITK-SNAP software. A) Region of interest ( ROI ) of mandibular condyles in multiplanar reconstructions in the ITK-SNAP software. B) Application of bubble in “edge attraction” mode, for semi-automatic segmentation, and the segmented condyle in yellow. C) 3D virtual model after segmentation.

In ITK-SNAP, the region of interest for the semi-automatic segmentation of both condyles was standardized from the most superior point of the condyle to the most inferior portion of the mandibular notch (Fig 2A and 2B), as described in a previous study.^25^ After segmentation, the 3D virtual models of both mandibular condyles were created, and the same software was used to calculate the volume in mm^3^ (Fig 2C).

### STATISTICAL ANALYSIS

To assess the intra and inter-examiner agreement, the intraclass correlation coefficient (ICC) was calculated. Assessment of the MA considered the side affected by the condition studied (UCLP), and the means and standard deviation between the cleft side (CS) and noncleft side (NCS) were compared. The linear and volumetric measurements of CS and NCS were compared using the paired *t-*test for dependent samples.

The means and standard deviation were evaluated using the Mann-Whitney test, to establish the chin deviation. Fisher’s exact test was used to assess the tendency and association between the deviation and the side affected by the cleft. Analyses were stratified by growth stage, into prepubertal and pubertal groups. The R software (The University of Auckland, New Zealand) was used, and a level of significance of 0.05 was adopted.

## RESULTS

After an initial analysis of the sample, 5 CBCT scans were excluded (2 presenting isolated cleft palate; 3 were in the post-pubertal stage), and a total of 47 CBCT scans of patients with UCLP were included in the final sample, consisting of 17 female (36%), and 30 male (64%), with chronological ages between 6 and 14 years (mean: 10.64 ± 2.04 years). In the prepubertal stage, 20 were male (64%), and 11 were female (36%), with chronological ages between 6 and 12 years (mean: 9.6±1.5 years). In the pubertal stage, 10 were male (62%), and 6 were female (38%), with chronological ages between 9 and 14 years (mean: 12.6±1.5 years), as presented in Table 1. The intraclass correlation coefficient (ICC) in the inter and intra-examiner ranged between 0.879-1 and 0.994-0.999, respectively, demonstrating excellent agreement.^26^

**Table 1:** Demographic features of the UCLP patients.

Variables	Total sample n = 47	Prepubertal n = 31 (66%)	Puberal n = 16 (34%)
Age (mean ± standard deviation)	10.64 ± 2.04 years	9.6 ± 1.5 years	12.6 ± 1.5 years
Sex - n (%)			
Male	30 (64%)	20 (64%)	10 (62%)
Female	17 (36%)	11 (36%)	6 (38%)
Cleft side - n (%)			
Right	16 (34%)	9 (29%)	5 (31%)
Left	31 (66%)	22 (71%)	11 (69%)

Mandibular linear measurements (mm), gonial angle, and the volume of mandibular condyles (mm^3^) in patients with UCLP on CS and NCS are shown in Table 2. In prepubertal patients, the Co-Me in the CS (105.5 ± 5.7 mm) was significantly (*p* = 0.036) smaller when compared with the NCS (106.03 ± 5.7 mm). In patients at the pubertal stage, the Co-Go and the Go-Me were significantly lower (46 ± 6 mm; *p* = 0.004) and higher (78.5 ± 5.8 mm; *p*= 0.026) in the CS, when compared with NCS (Co-Go= 49 ± 6 mm; Go-Me= 77.0 ± 64.6 mm). 

**Table 2:** Three-dimensional linear measurements, gonial angle, and volume of the mandibular condyles in UCLP patients in prepubertal and pubertal stages.

	Prepubertal			Pubertal		
Variable	CS^1^ (n = 31)	NCS^1^ (n=31)	p^2^	CS^1^ (n = 16)	NCS^1^ (n = 16)	p^2^
Co-Go	45.19 (3.27)	44.92 (3.09)	0.476	46 (6)	49 (6)	0.004*
Co-Me	105.5 (5.7)	106.3 (5.7)	0.036*	110.4 (5.3)	111.1 (5.8)	0.187
Go-Me	73.8 (5.9)	74.2 (6.0)	0.295	78.5 (5.8)	77.0 (4.6)	0.026*
CoGoMe	124.2 (5.0)	125.4 (4.4)	0.059	127.3 (6.9)	126.5 (5.5)	0.465
Volume (mm^3^)	1,171 (273)	1,176 (236)	0.875	1,204 (412)	1,213 (317)	0.873

¹ Mean (SD); ² Paired t-test; *Statistically significant association (*p* < 0.05). CS = cleft side. NCS = noncleft side.

The chin deviation values relative to the mandibular side (right or left) and the cleft side are shown in Tables 3 and 4. No statistically significant differences were observed in mean deviations values for the right (1.94 ± 1.39 mm) and left (1.63 ± 1.37 mm) sides (Table 3). However, a significant association (*p* < 0.001) between the deviated side and the cleft side was found (Table 3). At both growth stages, a statistically significant association was observed between the side to which the chin has deviated and the side on which the cleft lip and palate were present (prepubertal, *p*< 0.001; pubertal, *p* = 0.013) (Table 4).

**Table 3:** Mean and comparison between chin deviation and cleft side in UCLP patients.

	Chin deviation		p^3,4^
	Right (n =14)	Left (n = 33)	
	1.94 (1.39)^1^	1.63 (1.37)^1^	0.301^3^
			<0.001*^4^
Cleft side - Right	13 (93%)^2^	3 (9.1%)^2^	
Cleft side - Left	1 (7.1%)^2^	30 (91%)^2^	

^1^Mean (SD); ^2^n (%); ^3^Mann-Whitney test; ^4^Fisher’s exact test; *Statistically significant association (*p* < 0.05).

**Table 4:** Comparison between chin deviation and cleft side ( CS ) in UCLP patients in prepubertal and pubertal stages.

	Prepubertal			Puberal		
	Chin deviation		p^2^	Chin deviation		p^2^
	Right (n = 9)	Left (n = 22)		Right (n = 5)	Left (n = 11)	
CS			<0.001*			0.013*
Right	9 (100%)^1^	2 (9.1%)^1^		4 (80%)^1^	1 (9.1%)^1^	
Left	0 (0%)^1^	20 (91%)^1^		1 (20%)^1^	10 (91%)^1^	

^1^n (%); ^2^Fisher’s exact test; *Statistically significant association (*p* < 0.05).

## DISCUSSION

Some previous studies that evaluated MA in UCLP patients reported controversial results.^8^ ^,^ ^11^ Differences regarding sample size, chronological age or growth stages, in addition to the lack of standardization of the evaluation method,^5^ ^,^ ^8^ ^,^ ^9^ ^,^ ^11^ as well as to the fact that the mandible is not directly affected by the cleft, or to the multifactorial etiology of the mandibular asymmetry (genetic and environmental factors) could, in part, explain the controversy.^6^ ^,^ ^27^ ^,^ ^28^ In particular, environmental factors have been reported to impact the prevalence of malocclusions worldwide.^29^ ^,^ ^30^ In patients affected by UCLP, the presence of unilateral crossbite is generally observed, causing masticatory changes and asymmetric muscle function.^10^ The persistence of this malocclusion during craniofacial development, associated with the patient’s low socioeconomic level (environmental factor), often delays the orthodontic treatment, aggravating the asymmetrical pattern.

To the best of our knowledge, no previous studies evaluating MA in UCLP patients at different growth stages have been described in the literature. Therefore, the present study was conducted to address this issue. As in a previous study,^5^ we consider that the comparison between sides only in UCLP patients is sufficient to confirm the presence of a clinically relevant asymmetry. Comparison with a control group would possibly contribute to analyze the intensity of the asymmetry present in these patients; however, due to the lack of justification to obtain CBCT with a large field of view in pediatric patients without facial malformation or surgical indication, this comparison was not performed. 

In prepubertal stage patients, the Co-Me was significantly shorter in the CS, a result that was also observed in a previous study.^9^ However, other studies reported that Go-Me was also shorter in the CS.^5^ ^,^ ^9^ Sample characteristics, such as different cervical maturation stages^9^ and mean chronological age (22 year-old)^5^ in addition to the lack of information relative to the identification of landmarks in the methodology could in part explain this difference.

A previous study^4^ reported that the linear measurements of the Co-Go and Go-Me showed no differences between the CS and NCS of patients with UCLP with a mean age (14.32 years) that was higher than the mean age of patients in the pubertal stage in the present study. These previous results differed from those observed in the present study for the patients at the pubertal stage, in which the Co-Go and Go-Me measurements were significantly smaller in the CS, compared with the NCS. Again, sample organization, chronological age, and application of landmarks for measuring distances on three-dimensional virtual models built by volume rendering, instead of applying landmarks in the tomographic scan, can, in part, explain these differences.

Similar to a previous study,^10^ in the present investigation patients in the pubertal stage (CVMS3 and CVMS4; mean age = 12.5 years) had a lower Co-Go in the CS. The asymmetry in the UCLP patients in the pubertal stage was also characterized by a longer Go-Me in the CS. In contrast, a previous study^4^ reported no differences between the CS and NCS, and other studies did not observe a smaller dimension in the CS.^5^ ^,^ ^9^

A difference of more than 2 mm between the mandibular sides should be considered a clinically relevant asymmetry.^31^ In this regard, the Co-Go measurement in the pubertal stage in the present study exhibited clinical relevance since the mean difference was 3 mm, therefore, it was characterized as a moderate asymmetry.^31^

Contrasting with previous studies^9^ ^,^ ^11^, in which the greater CoGoMe was observed in the CS of UCLP patients, in the present study, no statistically significant differences between CS and NCS were observed for this measurement in patients at the prepubertal or pubertal stages. However, one previous study^9^ selected patients before pubertal growth spurts in only one group (CVMS1 to CVMS3), and the present study organized two groups (CVMS1 and CVMS2, as the prepubertal stage; CVMS3 and CVMS4, as the pubertal stage), similar to a previous study^23^, and another study^11^ organized the sample by chronological age and used different methods to apply the landmarks. 

Some studies have associated MA with volumetric alterations of the mandibular condyles.^5^ ^,^ ^32^ ^,^ ^33^ In the present study, as well as in a previous study,^4^ no statistically significant differences were observed between the volumes of the mandibular condyles in the CS and NCS in prepubertal and pubertal stages, which suggests that the mandibular condyles are not involved in the MA of patients with UCLP.

In the present study, a mean chin deviation of 1.63 mm to the left side and of 1.94 mm to the right side was observed; however, these values did not present statistical significance as well as clinical relevance, since the measurements observed were smaller than 4 mm.^34^ Nevertheless, in both growth stages a significant association was observed between the side to which the chin was deviated and the side on which the UCLP was present. Kim et al.^5^ and Lin et al.^9^ also observed that the chin tended to deviate towards the CS, with mean deviations of 1.59 mm and 1.63 mm, respectively. 

The results of the present study indicated that MA in individuals with UCLP in the prepubertal or pubertal stages must be considered when the treatment plan is elaborated. In this regard, the use of 3D technology is an important tool for identifying and evaluating the extent of craniofacial defects related to the CS. Moreover, orthodontic treatment interventions for correcting or minimizing the asymmetric mandibular growth would potentially improve facial esthetics and decrease the complexity of the orthognathic surgery to be performed after completion of growth in patients with UCLP.^5^

The present study evaluated MA in patients with UCLP using an accurate methodology^8^ in a cross-sectional design. Future studies with a prospective or longitudinal design, investigating the mandibular skeletal asymmetry in patients in prepubertal, pubertal and post-pubertal stages are necessary to improve the knowledge of the impact of these asymmetries on the patient’s occlusion and facial soft tissue characteristics.

## CONCLUSIONS

» Chin deviates towards the cleft side (CS) in both the prepubertal and pubertal growth stages.
» Unilateral cleft lip and palate (UCLP) patients in the prepubertal stage have a shorter Co-Me in the CS.
» UCLP patients in the pubertal stage have a lower Co-Go and higher Go-Me in the CS. The Co-Go in the pubertal stage showed clinical relevance, since it is characterized as moderate asymmetry, and it demonstrates the importance of monitoring mandibular growth.

## References

[1] Salari N, Darvishi N, Heydari M, Bokaee S, Darvishi F, Mohammadi M (2022). Global prevalence of cleft palate, cleft lip and cleft palate and lip a comprehensive systematic review and meta-analysis. J Stomatol Oral Maxillofac Surg.

[2] Nascimento VC, Martins MM, Vilella BS, Faco R, Timmerman H, De Clerk H (2023). Impact of bone-anchored maxillary protraction on the quality of life of subjects with complete unilateral cleft lip and palate. Am J Orthod Dentofacial Orthop.

[3] Shetye PR, Evans CA (2006). Midfacial morphology in adult unoperated complete unilateral cleft lip and palate patients. Angle Orthod.

[4] Golshah A, Hajiazizi R, Azizi B, Nikkerdar N (2022). Assessment of the asymmetry of the lower Jaw, face, and palate in patients with unilateral cleft lip and palate. Contemp Clin Dent.

[5] Kim KS, Son WS, Park SB, Kim SS, Kim YI (2013). Relationship between chin deviation and the position and morphology of the mandible in individuals with a unilateral cleft lip and palate. Korean J Orthod.

[6] Laspos CP, Kyrkanides S, Tallents RH, Moss ME, Subtelny JD (1997). Mandibular asymmetry in noncleft and unilateral cleft lip and palate individuals. Cleft Palate Craniofac J.

[7] Halicioglu K, Celikoglu M, Caglaroglu M, Buyuk SK, Akkas I, Sekerci AE (2013). Effects of early bilateral mandibular first molar extraction on condylar and ramal vertical asymmetry. Clin Oral Invest.

[8] Paknahad M, Shahidi S, Bahrampour E, Beladi AS, Khojastepour L (2018). Cone beam computed tomographic evaluation of mandibular asymmetry in patients with cleft lip and palate. Cleft Palate Craniofac J.

[9] Lin Y, Chen G, Fu Z, Ma L, Li W (2015). Cone-beam computed tomography assessment of lower facial asymmetry in unilateral cleft lip and palate and non-cleft patients with class III skeletal relationship. PLoS One.

[10] Celikoglu M, Halicioglu K, Buyuk SK, Sekerci AE, Ucar FI (2013). Condylar and ramal vertical asymmetry in adolescent patients with cleft lip and palate evaluated with cone-beam computed tomography. Am J Orthod Dentofacial Orthop.

[11] Kurt G, Bayram M, Uysal T, Ozer M (2010). Mandibular asymmetry in cleft lip and palate patients. Eur J Orthod.

[12] Starbuck JM, Ghoneima A, Kula K (2015). A multivariate analysis of unilateral cleft Lip and palate facial skeletal morphology. J Craniofac Surg.

[13] Jahanbin A, Eslami N, Hoseini Zarch H, Kobravi S (2015). Comparative evaluation of cranial base and facial morphology of cleft lip and palate patients with normal individuals in cone beam computed tomography. J Craniofac Surg.

[14] Nguyen T, Cevidanes L, Paniagua B, Zhu H, Koerich L, De Clerck H (2014). Use of shape correspondence analysis to quantify skeletal changes associated with bone-anchored Class III correction. Angle Orthod.

[15] Suri S, Prasad C, Tompson B, Lou W (2013). Longitudinal comparison of skeletal age determined by the Greulich and Pyle method and chronologic age in normally growing children, and clinical interpretations for orthodontics. Am J Orthod Dentofacial Orthop.

[16] Beit P, Peltomäki T, Schätzle M, Signorelli L, Patcas R (2013). Evaluating the agreement of skeletal age assessment based on hand-wrist and cervical vertebrae radiography. Am J Orthod Dentofacial Orthop.

[17] Karlberg J (2002). Secular trends in pubertal development. Horm Res.

[18] Delemarre-van de Waal HA (2005). Secular trend of the timing of puberty. Endocr Dev.

[19] McNamara JA, Franchi L (2018). The cervical vertebral maturation method a user's guide. Angle Orthod.

[20] Oenning AC, Jacobs R, Salmon B, DIMITRA Research Group (2021). ALADAIP, beyond ALARA, and towards personalized optimization for pediatric cone-beam CT. Int J Paediatr Dent.

[21] Vandenbroucke JP, von Elm E, Altman DG, Gøtzsche PC, Mulrow CD, Pocock SJ (2014). Strengthening the Reporting of Observational Studies in Epidemiology (STROBE) explanation and elaboration. Int J Surg.

[22] Carmo WD, Verner FS, Aguiar LM, Visconti MA, Ferreira MD, Lacerda MFLS (2021). Missed canals in endodontically treated maxillary molars of a Brazilian subpopulation prevalence and association with periapical lesion using cone-beam computed tomography. Clin Oral Investig.

[23] Franchi L, Baccetti T, McNamara JA (2000). Mandibular growth is related to cervical vertebral maturation and body height. Am J Orthod Dentofacial Orthop.

[24] Villoria EM, Souki BQ, Antunes FL, Andrade I, Oliveira DD, Soares RV (2023). Craniofacial morphology of patients with unilateral cleft Lip and palate at two stages of skeletal maturation. Braz Oral Res.

[25] Nawawi AP, Rikmasari R, Kurnikasari E, Oscandar F, Lita YA (2022). Volumetric analysis of normal condyles and those with disc displacement with reduction in the Indonesian population a CBCT study. Imaging Sci Dent.

[26] Rosado LPL, Barbosa IS, Junqueira RB, Martins APVB, Verner FS (2021). Morphometric analysis of the mandibular fossa in dentate and edentulous patients: a cone beam computed tomography study. J Prosthet Dent.

[27] Thiesen G, Gribel BF, Freitas MP (2015). Facial asymmetry a current review. Dental Press J Orthod.

[28] Ahn JS, Hwang HS (2001). Relationship between perception of facial asymmetry and posteroanterior cephalometric measurements. Korean J Orthod.

[29] Alhammadi MS, Halboub E, Fayed MS, Labib A, El-Saaidi C (2018). Global distribution of malocclusion traits: a systematic review. Dental Press J Orthod.

[30] Todor BI, Scrobota I, Todor L, Lucan AI, Vaida LL (2019). Environmental factors associated with malocclusion in children population from Mining Areas, Western Romania. Int J Environ Res Public Health.

[31] Ramirez-Yañez GO, Stewart A, Franken E, Campos K (2011). Prevalence of mandibular asymmetries in growing patients. Eur J Orthod.

[32] Tun Oo L, Miyamoto JJ, Takada JI, Cheng SE, Yoshizawa H, Moriyama K (2022). Three-dimensional characteristics of temporomandibular joint morphology and condylar movement in patients with mandibular asymmetry. Prog Orthod.

[33] Ikeda K, Kawamura A (2009). Assessment of optimal condylar position with limited cone-beam computed tomography. Am J Orthod Dentofacial Orthop.

[34] Haraguchi S, Takada K, Yasuda Y (2002). Facial asymmetry in subjects with skeletal Class III deformity. Angle Orthod.

